# Phages connect the biological dots of antimicrobial resistance: from genesis and spread to alternative treatment modules

**DOI:** 10.1093/jac/dkaf293

**Published:** 2025-08-06

**Authors:** Cleo Anastassopoulou, Antonios-Periklis Panagiotopoulos, Stefanos Ferous, Athanasios Tsakris

**Affiliations:** Department of Microbiology, Medical School, National and Kapodistrian University of Athens, 75 Mikras Asias Street, Athens 11527, Greece; Department of Microbiology, Medical School, National and Kapodistrian University of Athens, 75 Mikras Asias Street, Athens 11527, Greece; Department of Microbiology, Medical School, National and Kapodistrian University of Athens, 75 Mikras Asias Street, Athens 11527, Greece; Department of Microbiology, Medical School, National and Kapodistrian University of Athens, 75 Mikras Asias Street, Athens 11527, Greece

## Abstract

Antimicrobial resistance (AMR) poses a severe global health threat, necessitating alternatives to conventional antibiotics, which are increasingly ineffective. Phages play a dual role in both propagating and potentially mitigating AMR. They facilitate AMR dissemination primarily through transduction, with emerging evidence suggesting indirect involvement in conjugation and transformation. Phage–plasmids, a dynamic entity bridging phages and plasmids, have gained increasing attention for their role in AMR evolution. Conversely, phage therapy has demonstrated promise in targeting MDR pathogens and disrupting biofilms through lytic activity and enzymatic degradation. However, challenges, such as phage resistance, host specificity and regulatory constraints, must be addressed to enable widespread clinical implementation. While regulatory frameworks for phage therapy remain underdeveloped in many regions, initiatives such as the EMA workshop in 2015 have sought to establish pathways for regulatory approval, addressing issues related to phage standardization, phage production, quality control, clinical validation and product monitoring. Leveraging the extensive experience of Eastern European countries, where phage therapy has been successfully integrated into medical practice, may accelerate its acceptance in Western healthcare systems. Integrating phages with existing antimicrobial strategies may provide a viable approach to combating AMR. Phages thus connect the biological dots of AMR by contributing to its generation and spread, but possibly also to its resolution, likely in combination with antibiotics.

## Introduction

Antimicrobial resistance (AMR) represents one of the most significant global health challenges in modern medicine. We are gradually approaching an era devoid of effective antibiotics, where once-treatable common infections may prove fatal.^1^ The excessive misuse and overuse of antibiotics across various sectors, including human and veterinary medicine as well as agriculture, aquaculture and horticulture, have rendered AMR a paramount threat to all facets of medical treatment.^1^ The global burden of AMR is evident in WHO projections for 2050, which estimate that drug-resistant infections could directly cause ∼1.91 million deaths, with an additional 8.22 million deaths associated with AMR worldwide.^2^ Furthermore, the World Bank estimates that AMR could lead to increases amounting to $1 trillion in healthcare costs by 2050, further emphasizing its economic and public health implications.^2^ These alarming figures underscore the urgent need for alternative effective strategies to mitigate its impact.

The growing threat of AMR has exposed critical limitations in currently available antibiotic regimens.^3^ One of the most pressing concerns is the stagnation in antibiotic discovery, as no novel chemical classes or mechanisms of action have been identified in the past three decades.^3^ Compounding this issue, pharmaceutical companies have largely deprioritized antibiotic research due to financial constraints and significant developmental challenges.^3^ The difficulty of antibiotics to penetrate and destroy biofilms has further driven the search for alternative therapies.^4^ Cells within biofilms undergo intricate adaptations, including unique changes in gene expression and protein production, allowing them to endure extreme environmental conditions, hence greatly diminishing the effectiveness of antibiotics.^4^ While few potent antibiotics, that are frequently used in combination, remain available for severe infections, their use is often accompanied by a heightened risk of adverse effects.^4^ Given these limitations, the urgent need for alternative treatments has reignited the interest in bacteriophage therapy nearly a century after its initial clinical application.

Bacteriophages or phages are viruses that specifically infect bacteria and that play a crucial, but often underappreciated, role in microbial ecology and evolution.^5^ These viruses are among the most abundant entities in the biosphere and are believed to represent some of the earliest biological forms on Earth.^6^ The first documented observations of bacteriophages date back to 1915, when Frederick Twort reported an agent inhibiting the growth of bacterial in cultures.^7^ Two years later, in 1917, Félix d’Hérelle independently described a similar phenomenon and coined the term bacteriophage to describe these bacteria-destroying entities.^7^ Intrigued by their potential, d’Hérelle initiated pioneering efforts to explore bacteriophages as therapeutic agents, applying them in experimental treatments for bacterial infections as early as 1919.^8^ The first recorded clinical application followed in the USA in 1922 to treat dysentery.^9^ Although phage therapy held significant promise, the understanding of phage biology and the exact mechanisms of action of bacteriophages remained limited. As antibiotics began to emerge successfully, interest in phage therapy faded.^10^ Research on phages persisted primarily in the Soviet Union and other Eastern European countries, while the Cold War tensions and negative sentiments towards the Soviet Union further contributed to the declined interest in phage therapy in the USA.^11^

Phages replicate through lytic or lysogenic cycles. In the lytic cycle, they multiply and ‘lyse’ (destroy) the host cell relatively rapidly, while in the lysogenic cycle, they integrate into the bacterial genome and replicate passively for longer periods before switching to the lytic cycle.^12^ Virulent phages follow only the lytic cycle, whereas temperate phages can switch between both cycles.^12^ Temperate phages have a unique place in bacterial evolution, not only by integrating into host genomes as prophages but also by driving horizontal gene transfer (HGT), introducing genetic diversity and serving as competitive tools against rival strains.^13^ These roles assist bacterial adaptation to new environments and survival under changing conditions.^13^

Under the selective forces shaping bacterial evolution, bacteriophages play a particularly complex and seemingly paradoxical role. On one hand, phages contribute to the generation and spread of AMR by facilitating the horizontal transfer of resistance genes between bacteria, thereby accelerating the emergence of drug-resistant strains. On the other hand, these same viruses have re-emerged recently as a promising solution to the growing problem of AMR, offering a highly specific, inherently non-toxic and theoretically cost-effective approach to selectively targeting resistant bacterial pathogens. This dual nature of phages—as both contributors to the problem and a potential solution for AMR—raises important questions about how they can be safely harnessed for therapeutic use. Although their ability to spread resistance genes cannot be ignored, phage therapy remains a powerful tool in the fight against MDR infections, offering a path forward in an era where antibiotics are becoming increasingly obsolete.

## Phages as contributors to the problem of AMR

**Figure 1.**
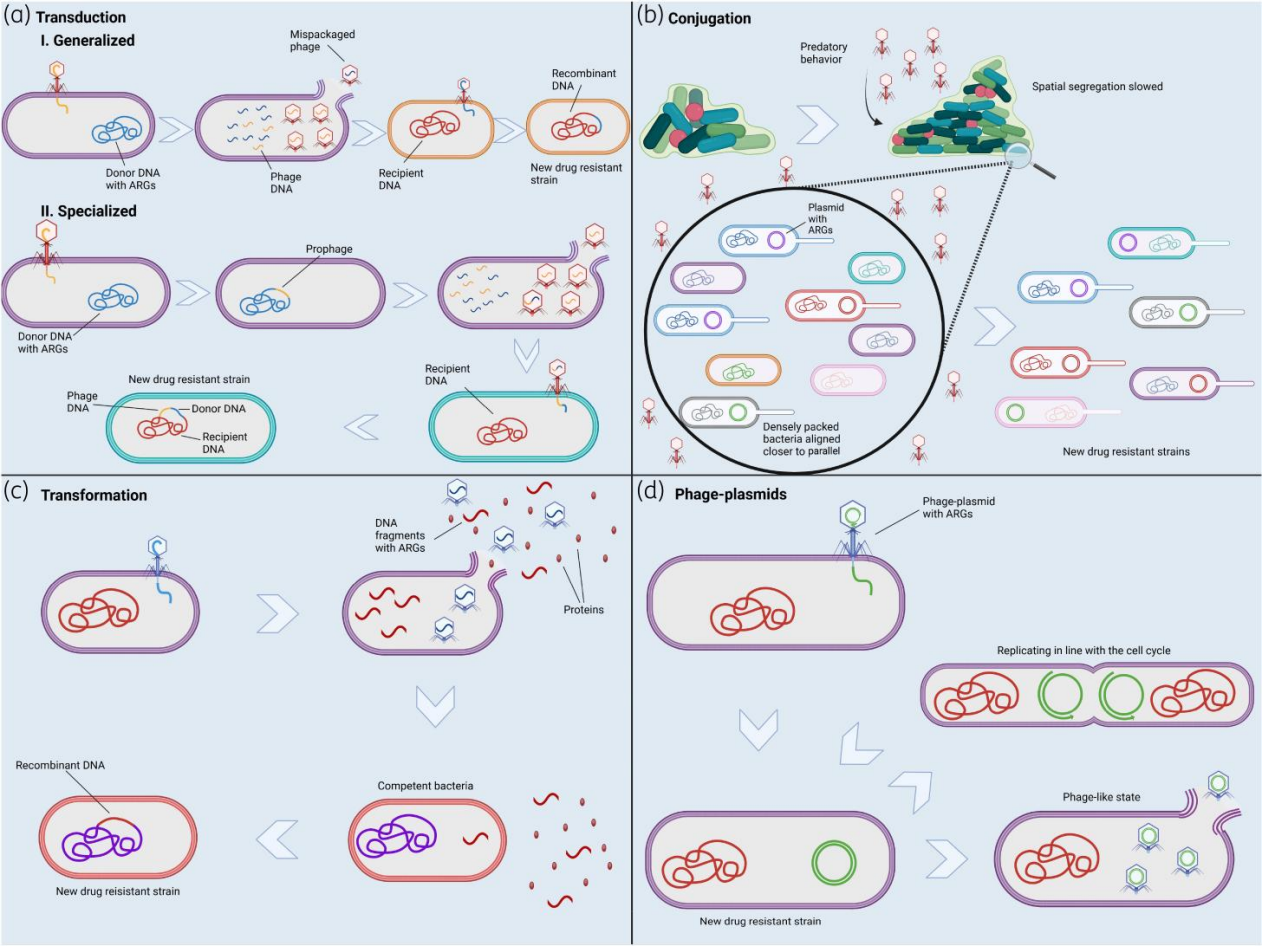
The involvement of phages in the generation and spread of AMR. (a) Transduction: (I) Generalized: A mispackaged phage transfers ARG-containing bacterial DNA to a new host. In addition, ARG-containing DNA can be transferred from plasmids. (II) Specialized: Phage DNA recombined with adjacent donor DNA containing ARGs gets integrated into a recipient genome. (b) Conjugation: Phage predation disrupts bacterial spatial organization, increasing cell–cell interactions and enhancing plasmid-mediated ARG transfer. (c) Transformation: Phage-induced bacterial lysis releases extracellular DNA fragments with ARGs, which can be taken up by competent bacteria. (d) Phage–plasmids: These elements replicate alongside the host genome but can transition into a phage-like state, further disseminating ARGs (created with BioRender.com).

Bacteriophages play a crucial role in bacterial evolution by facilitating HGT, a key process in genetic adaptation.^14^ Although phages have been widely recognized for their antimicrobial potential, their role in the spread of AMR is starting to get recognized.^14^ Phages can transfer antibiotic resistance genes (ARGs) between bacterial populations, accelerating the emergence and dissemination of MDR strains.^14^ This process, primarily mediated by transduction, underscores the complex interactions between phages and bacteria and highlights the unintended consequences of phage activity in both clinical and environmental settings.^14^ The involvement of phages in the genesis and spread of AMR is illustrated in Figure 1.

### Phage-mediated transduction

Among the three mechanisms of HGT—conjugation, transformation and transduction—phage-mediated transduction plays a particularly important role in the horizontal spread of ARGs.^15^ In microbiology, transduction is categorized as either generalized or specialized, depending on the mechanism by which genetic material is transferred between bacteria.^15^ Generalized transduction can occur in both virulent and temperate phages during the lytic cycle.^15^ In this process, bacterial genes are transferred between cells due to errors in viral packaging, when phages mistakenly incorporate fragments of host DNA, including ARGs, instead of their own genetic material.^15^ In some cases, this mispackaged DNA may include small plasmids carrying ARGs, which can be transduced as concatemers and later resolved in the recipient cell.^16^ For example, during generalized transduction mediated by phage P22 in *Salmonella enterica*, plasmid DNA can be mistakenly packaged into phage particles and delivered into recipient cells.^16^ In *Staphylococcus aureus*, phage ϕ11 has been shown to transduce small plasmids as linear concatemers generated through plasmid-initiated replication.^17^ When these mispackaged viral particles infect a new bacterial host, the donor DNA can be integrated into the recipient’s genome, facilitating the horizontal transfer of AMR.^15^ In cases where entire plasmids are transferred, the plasmid DNA can also persist in the recipient cell as an extrachromosomal element rather than integrating into the genome.^18^ Specialized transduction is a process exclusive to temperate phages during the lysogenic cycle.^19^ It occurs when a prophage undergoes improper excision from the host genome, unintentionally capturing adjacent bacterial genes.^19^ As a result, a limited set of host genes is transferred to a new bacterial cell upon infection, contributing to genetic variation and potentially the spreading of AMR.^19^

### Phage involvement in conjugation

Apart from transduction, conjugation is another mechanism through which phages contribute to the spread of AMR. Ruan *et al*.^20^ have demonstrated recently that phage predation enhances plasmid-mediated gene transfer by disrupting bacterial colony organization. By preferentially targeting cells at the periphery of growing bacterial populations, phages prevent natural separation, increasing direct cell-to-cell contact.^20^ This, in turn, facilitates the horizontal transfer of ARGs through conjugation, even in the absence of selective antibiotic pressure.^20^ These findings underscore the notion that, beyond transduction, phages can indirectly accelerate AMR dissemination by promoting plasmid exchange between bacterial cells.

### Phage HGT through transformation

Although bacteriophages are well known for spreading AMR through transduction, we hypothesize that they may also contribute to AMR dissemination indirectly via transformation, a process in which bacteria acquire free extracellular DNA.^21^ When a lytic phage infects a bacterial cell, it causes cell lysis, releasing chromosomal fragments and plasmids, some of which may carry ARGs.^21^ Under favourable conditions, naturally competent bacteria, such as *Acinetobacter* spp., *Streptococcus pneumoniae* and *Neisseria* spp., can take up these DNA fragments from the environment and integrate them into their genomes through homologous recombination.^21^ This mechanism, although theoretically possible, is rarely discussed as a major route of phage-driven AMR transfer. Phage-induced bacterial lysis could increase the local concentration of free DNA, rendering transformation-mediated AMR spread more likely under specific conditions, such as in biofilms.

### Phage–plasmids

Phage–plasmids have recently attracted significant attention for their role in bacterial evolution and HGT.^22^ These mobile genetic elements exhibit characteristics of both plasmids and temperate phages, allowing them to function in multiple genetic contexts.^22^ Compared with conventional phages or plasmids, phage–plasmids possess larger genomes, incorporating genetic elements homologous to both, with their composition varying depending on the prevailing environmental conditions in which they are found.^22^ Studies have shown that phage–plasmids frequently carry ARGs and facilitate their transfer between bacterial populations.^23^ They function as plasmids by acquiring genetic material from other plasmids, while their role as phages enables them to infect diverse bacterial species, facilitating the further spread of resistance genes.^23^ It has been observed that these temperate phages function as extrachromosomal plasmids within the host genome, replicating in synchrony with the bacterial cell cycle.^22^ The exact impact phage–plasmids have on AMR spread is not yet determined and ongoing research is increasingly focused on understanding their impact.^23^

### Lysogeny and ARG acquisition

Likewise, aside from the other described mechanisms by which phages contribute to AMR, prophages can serve as reservoirs for resistance genes by acquiring foreign DNA during the lysogenic phase.^24^ This often involves the insertion of mobile genetic elements, such as insertion sequences, into the prophage region of the bacterial genome.^24^ For example, in *Streptococcus suis*, the resistance gene *optrA*, which confers resistance to oxazolidinones and phenicols, was found embedded within a prophage in one of the analysed isolates.^25^ This gene was flanked by two copies of the insertion sequence IS*1216* in the same orientation, forming a mobile element capable of excision and circularization.^25^ The study confirmed that the prophage-resident *optrA* locus could potentially mobilize and integrate into other genetic contexts, highlighting prophages as reservoirs for resistance genes even during lysogeny.^25^

## Phage therapy as a solution to the problem of AMR

Phage therapy is increasingly recognized as a promising alternative or adjunct to conventional antibiotics, particularly for combating MDR bacteria. Advances in phage engineering, synthetic biology and combination therapies have significantly enhanced its therapeutic potential. However, despite these developments, several challenges must be overcome to enable widespread clinical implementation.

### Advantages of phage therapy in the post-antibiotic era

Phage therapy is one of the most promising tools for fighting MDR bacteria in the post-antibiotic era. One of the key advantages of using phages to combat bacterial infections over conventional antibiotics is their high specificity.^26^ Unlike antibiotics that often eliminate both pathogenic and beneficial microbes, phages typically infect only a narrow range of bacterial strains.^26^ This specificity is driven by the coevolution of phages and their bacterial hosts, resulting in a highly selective recognition system that enables phages to target specific bacterial surface receptors.^26^ Consequently, phage therapy exerts minimal impact on the broader microbiota, lowering the risk of unintended side effects.^27^ Phages have demonstrated a favourable safety profile in both animal and clinical studies, with minimal reported adverse effects.^28^ Clinical trials and case reports have shown that phage therapy is generally well tolerated, with rare occurrences of mild inflammatory responses, primarily linked to bacterial lysis and endotoxin release.^28^

Moreover, because phages replicate only in the presence of their target bacteria, their activity remains self-regulating, reducing the chances of off-target effects and immune system activation.^29^ As long as bacterial hosts are present, phages continue to self-replicate, enhancing their therapeutic efficacy.^29^ Conversely, once the target bacteria are eliminated by phage activity, phage replication ceases, leading to their natural decline, thereby ensuring a self-limiting treatment approach.^29^

Biofilms, as mentioned above, pose a significant challenge to antibiotic therapies, as they serve as a key mechanism of bacterial resistance.^30^ These highly structured microbial communities, consisting of cells in diverse physiological and morphological states, adhere irreversibly to both biotic and abiotic surfaces.^30^ Bacteria within biofilms exhibit up to 1000-fold greater resistance to conventional antibiotics and host immune responses compared with their planktonic counterparts.^30^ Phage therapy presents a promising solution, as bacteriophages can target and disrupt biofilms through multiple mechanisms.^31^ However, we note that the inability of phages to replicate in the subpopulation of biofilm-associated bacteria that exhibit low or absent metabolic activity may limit the benefits of phage therapy against biofilms.^32^ One of the most effective strategies involves the production of specialized enzymes, such as depolymerases and lysins, which degrade the protective extracellular matrix, weakening the biofilm structure and enhancing bacterial clearance.^31^ These strategies have shown efficiency even against bacteria with low metabolic rates. Recent research showed that the endolysin LysSYL derived from a *Staphylococcus* phage significantly reduced bacterial load in pre-formed *S. aureus* biofilms, including MDR strains.^33^ Its biofilm-disrupting activity was linked to enzymatic cleavage of the peptidoglycan layer, compromising bacterial cell integrity within the matrix.^33^ Another study demonstrated that phage 168, which infects carbapenem-resistant *Klebsiella pneumoniae*, encodes depolymerase Depo168, that effectively degrades the bacterial capsule and disrupts biofilms.^34^ The enzyme reduced biofilm biomass and enhanced bacterial susceptibility to antibiotics and immune responses.^34^ Most experimental setups showed that although phage therapy was very efficient in combating biofilms, complete eradication was almost impossible. These enzymatic activities allow phages to penetrate and disrupt biofilms more effectively, especially in combination with antibiotics.^30^

In addition to systemic applications, topical phage therapy has shown promise, particularly for treating skin and soft tissue infections, surgical wounds and burns, settings where bacterial biofilms often present a significant treatment challenge.^35^ Topical administration enables direct, localized delivery of high phage concentrations to the infection site, thereby limiting systemic exposure and potentially reducing immune clearance.^35^ For instance, phage Pɸ-Bw-Ab demonstrated strong antibacterial activity against an XDR *Acinetobacter baumannii* strain isolated from burn wound infections in hospitalized patients, underscoring its potential in targeted phage therapy.^36^ Similarly, in a case of chronic osteomyelitis in a 60-year-old woman with long-standing *S. aureus* infection and Type 2 diabetes, a combination of topical and oral bacteriophage therapy led to wound closure within 18 weeks and sustained remission without antibiotics for over 2 years.^37^

### Successes of phage therapy thus far

Phage therapy has shown promising, albeit limited, results for the treatment of MDR bacterial infections, particularly against Gram-negative pathogens, such as *K. pneumoniae*, *Pseudomonas aeruginosa* and *A. baumannii.*^38^ Preclinical studies and isolated clinical cases have shown that bacteriophages can effectively reduce bacterial loads, improve survival rates in infected hosts and even reverse antibiotic resistance in some instances.^38^ In addition to their direct bactericidal activity, phages can weaken bacterial virulence by altering cell walls and membrane structures, rendering pathogens more susceptible to conventional antibiotics and immune clearance.^38^ Furthermore, phage–antibiotic combination therapies have been reported to exhibit synergistic effects, enhancing the overall efficacy of antimicrobial treatment.^31^ In a study targeting mature biofilms formed by resistant *K. pneumoniae*, the combination of pre-adapted bacteriophages and antibiotics significantly reduced biofilm biomass *in vitro*, achieving greater efficacy than either treatment alone.^39^ In experiments targeting MRSA, the combination of a staphylococcal phage with antibiotics showed strong synergy and significantly enhanced treatment efficacy.^40^  *In vivo*, this approach improved survival in infected larvae by up to 80%.^40^ However, it is important to note that such synergistic effects of phage/antibiotics combinations are often highly dependent on the specific *in vitro* experimental conditions, which may not fully replicate in clinical settings. Despite these promising results, the clinical application of phage therapy remains limited, with only a few randomized controlled trials available.^38^

### FDA-approved phage therapy applications

The extensive use of antibiotics in agriculture and livestock farming has played a major role in the emergence and spread of AMR. In many cases, antibiotics are not only administered for treating infections but also for promoting growth and preventing disease outbreaks.^41^ However, this practice creates selective pressure that drives the evolution of resistant bacterial strains, which can then spread to humans through food consumption, direct contact with animals or environmental contamination.^41^ Research has shown that reducing antibiotic use in food-producing animals can significantly lower the prevalence of resistant bacteria in these animals by up to 39%.^42^ Although measures have been implemented to reduce antibiotic usage, estimates suggest that antibiotic consumption in animal agriculture will rise by 8% between 2020 and 2030, further intensifying concerns about AMR.^43^

To address these concerns, the US FDA has approved several phage therapy products for use in food applications. Since 2006, phage-based interventions have been utilized to target foodborne pathogens such as *Listeria monocytogenes*, *Escherichia coli* O157:H7 and *Salmonella* spp. in meat and poultry processing.^44–46^ In 2017, the FDA granted authorization to a five-phage cocktail specifically targeting *Shigella* spp., following studies demonstrating its ability to reduce *Shigella* levels by ∼1 log in various food products.^47^ Phage preservation approaches have also been explored for application in fresh produce, with a focus on mitigating bacterial contamination and enhancing shelf life.^48^ In Europe, the European Food Safety Authority evaluated the safety and efficacy of phage products for the reduction of *L. monocytogenes* on various ready-to-eat food products and recommended their use.^49^

These phage treatments, classified as generally recognized as safe (GRAS), offer an effective and natural way to reduce bacterial contamination without the drawbacks associated with antibiotics.^45,46^ Several recent studies have explored the optimization and expansion of phage therapy in other applications like raw milk and cheese.^50^ Transitioning from antibiotics to phage therapy in the agricultural and livestock settings offers a promising strategy to mitigate the spread of AMR. A concerted effort to transition away from antibiotics in food production, agriculture and livestock farming, prioritizing phage therapy instead, could help preserve the effectiveness of antibiotics for human medical applications, where they are most critically needed.

### The need for a regulatory framework for phage therapy

Currently, there is no standardized regulatory framework governing the use of phages in combating AMR.^51,52^ Existing legislation generally requires a detailed qualitative and quantitative assessment of each component within a medicinal product, which poses challenges for the approval of phage-based therapies.^52^ In the USA, the FDA has yet to establish a dedicated approval pathway for phage therapy, although it has permitted its use under emergency authorizations and expanded access programmes for patients with MDR infections. Phage therapy is classified as an investigational new drug, allowing its use in human clinical trials.^53^ However, under this designation, rigorous evaluation is required to establish the safety and efficacy of phage-based treatments before they can be considered for broader clinical application.^54^

In the EU, phage therapy is regulated as a biological medicinal product under Directive 2001/83/EC.^55^ This classification means that phage-based treatments generally require marketing authorization, which entails submitting extensive pharmaceutical, preclinical and clinical data to confirm their safety, efficacy and quality.^55^ However, certain exceptions apply, including the use of phages in clinical trials, named-patient programmes and compassionate use cases.^55^ Some European countries have introduced national frameworks for phage therapy. Belgium, for instance, has developed a structured and modern regulatory framework for phage therapy. In response to discussions within the Belgian Chamber of Representatives in 2016 regarding the benefits and challenges of phage therapy regulation, the Belgian Federal Agency for Medicines was tasked with creating systematic guidelines.^56^ This led to the adoption of a magistral preparation approach, allowing pharmacists to produce phage-based treatments according to a physician’s prescription while adhering to pharmaceutical standards.^56^ Unlike many other EU countries, such as France and Germany, where compassionate use is restricted to emergency cases, Belgium’s magistral preparation framework enables personalized phage therapy for individual patients.^56^

In contrast, Eastern European nations, especially Georgia and Russia, have a longstanding tradition of clinically applied phage therapy, with several approved phage therapy products in use. A list of commercially available phage products for humans in Eastern European countries is presented in summary in Table 1.^57–71^ The regulatory frameworks in these countries differ significantly from those in the EU, and for that reason, they have faced criticism due to the insufficient procurement of antimicrobial data, unclear production processes and inadequate quality control in the development of phage cocktails.^56^ In Georgia, both pre-prepared and personalized phage medicines are classified as pharmaceuticals.^72^ Pre-prepared phage medicines require market authorization, while personalized phage treatments can be produced in specially licensed pharmacies through magistral preparation.^72^ Conversely, in Russia, although phage products are similarly categorized, personalized phage therapy is prohibited.^73^ The State Register of the Ministry of Health of the Russian Federation has authorized only NPO Microgen to manufacture commercial phage cocktails.^73^

**Table 1.** List of commercially available phage products for humans in Eastern European countries.

Country	Company	Product	Application
Czech Republic	MB Pharma	Lyzodol^®57^	Respiratory infections caused by *Propionibacterium acnes*, *Lelliottia amnigena*, *S. aureus* and *K. pneumoniae*
Georgia	Biochimpharm^58^	Phagyo^®^ spray Septaphage^®^ table Septaphage Phagyo^®^ PhageStaph Travelphag^™^	Prevention and treatment of pyo-inflammatory infections from multiple bacteria
	Eliava BioPreparation	Phago-FERSISI^59^	Pyo-inflammatory and enteric infections caused by *Staphylococcus* and *Streptococcus*
		Phago-Staph^60^	Infections caused by *Staphylococcal* bacteria
		Phago-Pyo^61^	Lysis of bacteria *Staphylococcus*, *Streptococcus*, *E. coli*, *Pseudomonas aeruginosa* and *Proteus*
		Phago-Intesti^62^	Lysis of bacteria *Shigella*, *Salmonella*, *E. coli*, *Proteus*, *Staphylococcus*, *Pseudomonas aeruginosa* and *Enterococcus*
		Phago-SES^63^	Pyo-inflammatory and enteric infections caused by *Staphylococcus*, *E. coli* and *Streptococcus*
		Phago-ENKO^64^	Enteric infections caused by *Staphylococcus*, *E. coli*, *Shigella* and *Salmonella*
Russia	Microgen	*Salmonella* Groups A, B, C and D bacteriophage^65^	*Salmonella*-related diseases
		*E. coli–Proteus* bacteriophage^65^	Enteric and inflammatory diseases, dysbacteriosis caused by *Proteus* and enterotoxigenic *E. coli*
		*Klebsiella*-purified polyvalent bacteriophage^65^	*K. pneumoniae*, *K. odorifera* and *K. rhinosclerosis*
		Dysentery polyvalent bacteriophage^65^	Bacterium that causes bacillary dysentery
		Complex pyobacteriophage^65^	*E. coli*, *K. pneumoniae*, *Streptococcus*, *Enterococcus*, *Proteus* and *K. pneumoniae*
	MicroMir	Phagogyn^66^	Protection against reproductive system bacterial infections
		Phagodent^67^	Balance oral microbiota
		Phagoderm^68^	Prevention of bacterial skin infections
		Otophagus^69^	Protection of the ear, nose and throat against bacterial and suppurative inflammation
		Iskraphage^70^	Normalization of the skin microbiota
Ukraine	Phagex	Pyofag^®71^	Pyo-inflammation and intestinal diseases caused by *P. aeruginosa*, *Proteus vulgaris*, *Proteus mirabilis*, *Streptococcus pyogenes*, *S. aureus* and *E. coli*
		Intestifag^®^ polyvalent bacteriophage^71^	*Shigella*, *Salmonella*, *E. coli*, *P. aeruginosa*, *Enterococcus faecalis* and *S. aureus*-related intestinal diseases.

Adapted from Karn *et al*.^54^

While phage therapy holds significant potential, several challenges must be addressed before its widespread adoption. Key barriers include the variability of phage preparations, the need for standardized manufacturing processes and unresolved intellectual property concerns. Unlike conventional antibiotics, phage therapy often requires individualized formulations, complicating regulatory standardization. Addressing these challenges requires international collaboration, with regulatory agencies developing adaptive frameworks that account for the unique properties of phages. In this context, the EMA convened a workshop in June 2015, bringing together experts from academia, industry and regulatory bodies to explore regulatory pathways for phage therapy. Discussions focused on establishing phage libraries, differentiating between personalized and standardized phage treatments and designing a flexible approval process that allows for necessary modifications to specific phage compositions.^55^ Leveraging the extensive experience and expertise of Eastern European countries, where phage therapy has been successfully implemented for decades, could facilitate its integration into Western medical practice. Collaborative efforts, including well-structured clinical trials, are essential to generating the scientific evidence needed for regulatory approval. Furthermore, establishing stringent quality control measures will help ensure the consistency, safety and efficacy of phage products. By addressing these regulatory and scientific challenges, phage therapy could become a viable alternative or supplement to antibiotics, playing a crucial role in combating AMR.

### Challenges and future research directions

As previously discussed, phage therapy offers several advantages in combating microbial infections, particularly those caused by MDR bacteria. While the high specificity of phages is a key factor contributing to their effectiveness against AMR, it also presents challenges for clinical implementation.^51^ The targeted nature of phage therapy necessitates the identification of a suitable phage that can effectively infect a particular bacterial strain, especially in personalized phage preparations tailored to specific clinical isolates.^51^ In a clinical setting, this requires isolating and characterizing the pathogen responsible for the infection before an appropriate therapeutic phage can be selected.^51^ This additional step can delay treatment, limiting the widespread application of phage therapy in urgent medical cases. Therefore, future research should prioritize the refinement of rapid and efficient diagnostic techniques for detecting and quantifying bacterial pathogens in clinical environments, enabling more timely and effective therapeutic interventions. Alternative strategies include the use of phage cocktails capable of targeting a broader range of bacterial strains or engineered phages designed to extend host range or enhance antibacterial activity.^74^

Recently, phage–lysin therapy has gained traction as a promising antimicrobial approach that harnesses enzymes produced by bacteriophages to break down bacterial cell walls.^75^ These lysins act rapidly and with high specificity, particularly against Gram-positive pathogens, leading to immediate bacterial cell lysis upon contact.^76^ Their modular structure, typically consisting of a catalytic and a binding domain, enables targeted activity, without disrupting beneficial bacteria.^75^ Due to their unique mode of action, lysins are less likely to induce resistance compared with traditional antibiotics, making them attractive candidates in the fight against MDR infections.^75^ The first clinical trial evaluating a phage–lysin therapeutic (NCT03163446) was concluded in 2020 and yielded encouraging results.^77^ Exebacase, a novel lysin specifically targeting *S. aureus*, demonstrated favourable tolerability and was associated with improved clinical outcomes in patients with *S. aureus* bloodstream infections when administered as an adjunct to standard therapy.^77^ Another recent study showed that combining the endolysin Ply2660 with the antimicrobial peptide LL-37 enhanced killing of drug-resistant *Enterococcus faecalis*, effectively disrupting biofilms and reducing infection severity *in vivo*.^78^

A major challenge in phage therapy is the potential for bacteria to develop resistance to phages, which could limit the long-term success of this approach. To defend against phage infections, bacteria have evolved various mechanisms, including altering or eliminating phage-binding receptors to prevent attachment, blocking the injection of phage DNA through specialized exclusion systems and degrading foreign genetic material using restriction–modification enzymes or the CRISPR-Cas adaptive immune system.^79^ One of the most effective strategies to overcome bacterial resistance to phages is the use of phage cocktails, which consist of multiple phages targeting different bacterial receptors, thereby delaying—if not reducing the likelihood—of resistance development.^80^ Combination therapies that integrate both phages and antibiotics have shown promise in addressing bacterial AMR by exploiting multiple mechanisms of bacterial eradication.^80,81^ These approaches can enhance treatment efficacy and help mitigate the emergence of phage-resistant bacterial strains.

An additional challenge of phage product development is the substantial associated financial cost.^82^ Although phage therapy is considered GRAS, it must still undergo rigorous clinical trials to demonstrate efficacy and safety prior to commercial use, a requirement that is unavoidable for any pharmaceutical product.^82^ However, the high cost of conducting these trials in the current changing socio-economic landscape can discourage manufacturers from investing given the financial risks involved.^82^ Engineering phages to address the limitations of WT strains can add to development costs, thereby presenting an additional barrier to the widespread implementation of the usage of phages for therapy.^82^

Although phage therapy is widely regarded as a relatively safe approach for combating MDR bacterial infections, its clinical application remains limited, with few trials conducted. Both animal studies and clinical trials have reported low toxicity and minimal adverse effects.^28^ However, due to the lack of extensive real-world implementation, the potential interactions between specific phages and the human immune system remain poorly understood. For instance, picobirnaviruses (PBVs) were long believed to be animal-infecting viruses, but recent evidence suggests they are actually prokaryotic RNA viruses.^83^ Notably, PBVs have been linked to graft-versus-host disease, yet their precise role in disease development remains unclear.^84^ Another important consideration in the safety of phage production is the removal of endotoxins, which are harmful bacterial components commonly present in lysates derived from Gram-negative hosts.^85^ Efficient purification methods have been shown to significantly reduce endotoxin levels, making phage preparations safer for clinical application.^85^ Given these uncertainties, further research should focus on elucidating phage–human immune interactions to ensure the long-term safety and viability of phage therapy in clinical settings.

**Figure 2.**
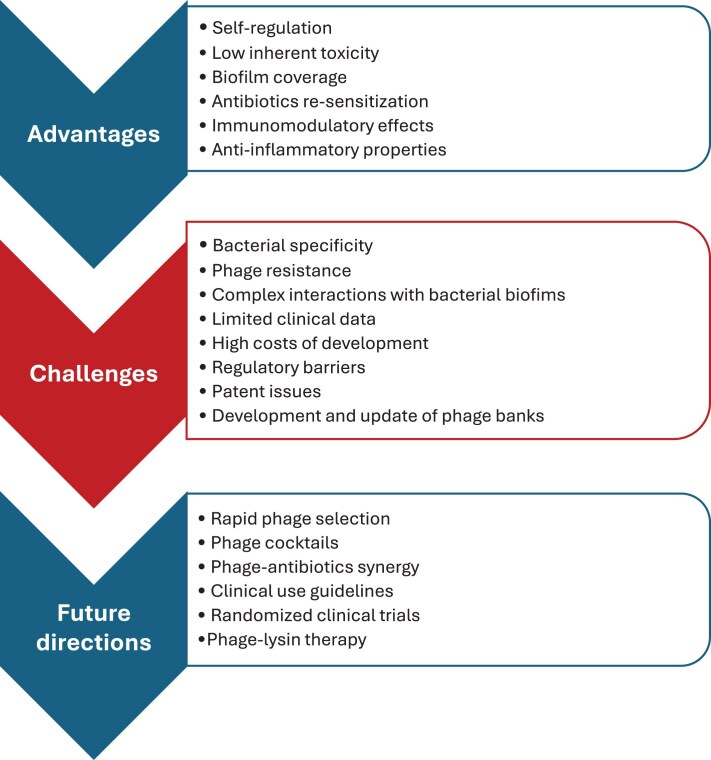
Advantages, challenges and future directions of phage therapy.

The advantages, challenges associated with its application and future directions of phage therapy are presented in Figure 2.

## Conclusion

Bacteriophages embody a biological paradox, serving as both the architects and potential demolishers of AMR. As drivers of HGT, they have facilitated the spread of ARGs, marking the beginning of AMR. Yet, as antibiotics lose effectiveness, phages re-emerge as a highly specific, self-replicating alternative or supplementary treatment, positioning them as the end of AMR, if harnessed correctly. This dual role presents both risks and opportunities. While phage therapy has shown promise against MDR bacteria, its clinical application presents challenges, including host specificity, bacterial resistance and regulatory uncertainty. Advances in synthetic biology, phage–antibiotic synergy and rapid bacterial identification are essential to overcoming these barriers. Ultimately, phages are neither inherently beneficial nor harmful—their impact depends on how we control and apply them. If guided by scientific innovation, they may serve as the final tool to counteract AMR, transforming them from contributors to the ultimate solution in the fight against resistant infections.

## References

[1] Krishnaprasad VH, Kumar S. Antimicrobial resistance: an ultimate challenge for 21st century scientists, healthcare professionals, and policymakers to save future generations. J Med Chem 2024; 67: 15927–30. 10.1021/acs.jmedchem.4c02002 39238216

[2] Antimicrobial resistance. n.d. https://www.who.int/news-room/fact-sheets/detail/antimicrobial-resistance.

[3] Balcázar JL . Implications of bacteriophages on the acquisition and spread of antibiotic resistance in the environment. Int Microbiol 2020; 23: 475–9. 10.1007/s10123-020-00121-5 32002743

[4] De la Fuente-Nunez C, Cesaro A, Hancock REW. Antibiotic failure: beyond antimicrobial resistance. Drug Resist Updat 2023; 71: 101012. 10.1016/j.drup.2023.101012 PMC12224857 37924726

[5] Debroas D, Siguret C. Viruses as key reservoirs of antibiotic resistance genes in the environment. ISME J 2019; 13: 2856–67. 10.1038/s41396-019-0478-9 31358910 PMC6794266

[6] Rohde C, Wittmann J. Phage diversity for research and application. Antibiotics 2020; 9: 734. 10.3390/antibiotics9110734 33114578 PMC7693090

[7] Salmond GPC, Fineran PC. A century of the phage: past, present and future. Nat Rev Microbiol 2015; 13: 777–86. 10.1038/nrmicro3564 26548913

[8] Summers WC . Bacteriophage therapy. Annu Rev Microbiol 2001; 55: 437–51. 10.1146/annurev.micro.55.1.437 11544363

[9] Davison WC . The bacteriolysant therapy of bacillary dysentery in children: therapeutic application of bacteriolysants; D’herelle’s phenomenon. Am J Dis Child 1922; 23: 531–4. 10.1001/archpedi.1922.01910420062011

[10] Ahmad TA, Houjeiry SE, Kanj SS et al From forgotten cure to modern medicine: the resurgence of bacteriophage therapy. J Glob Antimicrob Resist 2024; 39: 231–9. 10.1016/j.jgar.2024.10.259 39486687

[11] Summers WC . The cold war and phage therapy: how geopolitics stalled development of viruses as antibacterials. Annu Rev Virol 2024; 11: 381–93. 10.1146/annurev-virology-100422-040919 38848607

[12] Olszak T, Latka A, Roszniowski B et al Phage life cycles behind bacterial biodiversity. Curr Med Chem 2017; 24: 3987–4001. 10.2174/0929867324666170413100136 28412903

[13] Harrison E, Brockhurst MA. Ecological and evolutionary benefits of temperate phage: what does or doesn’t kill you makes you stronger. BioEssays 2017; 39: 1700112. 10.1002/bies.201700112 28983932

[14] Lerminiaux NA, Cameron ADS. Horizontal transfer of antibiotic resistance genes in clinical environments. Can J Microbiol 2019; 65: 34–44. 10.1139/cjm-2018-0275 30248271

[15] Zhang Y, Guo Y, Qiu T et al Bacteriophages: underestimated vehicles of antibiotic resistance genes in the soil. Front Microbiol 2022; 13: 936267. 10.3389/fmicb.2022.936267 35992716 PMC9386270

[16] Fillol-Salom A, Alsaadi A, de Sousa JAM et al Bacteriophages benefit from generalized transduction. PLoS Pathog 2019; 15: e1007888. 10.1371/journal.ppat.1007888 31276485 PMC6636781

[17] Novick RP, Edelman I, Lofdahl S. Small *Staphylococcus aureus* plasmids are transduced as linear multimers that are formed and resolved by replicative processes. J Mol Biol 1986; 192: 209–20. 10.1016/0022-2836(86)90360-8 2951524

[18] Cury J, Oliveira PH, De La Cruz F et al Host range and genetic plasticity explain the coexistence of integrative and extrachromosomal mobile genetic elements. Mol Biol Evol 2018; 35: 2230–9. 10.1093/molbev/msy123 29905872 PMC6107060

[19] Calero-Cáceres W, Ye M, Balcázar JL. Bacteriophages as environmental reservoirs of antibiotic resistance. Trends Microbiol 2019; 27: 570–7. 10.1016/j.tim.2019.02.008 30905524

[20] Ruan C, Ramoneda J, Kan A et al Phage predation accelerates the spread of plasmid-encoded antibiotic resistance. Nat Commun 2024; 15: 5397. 10.1038/s41467-024-49840-7 PMC11208555 38926498

[21] Johnsborg O, Eldholm V, Håvarstein LS. Natural genetic transformation: prevalence, mechanisms and function. Res Microbiol 2007; 158: 767–78. 10.1016/j.resmic.2007.09.004 17997281

[22] Pfeifer E, Moura de Sousa JA, Touchon M et al Bacteria have numerous distinctive groups of phage–plasmids with conserved phage and variable plasmid gene repertoires. Nucleic Acids Res 2021; 49: 2655–73. 10.1093/nar/gkab064 PMC7969092 33590101

[23] Pfeifer E, Bonnin RA, Rocha EPC. Phage–plasmids spread antibiotic resistance genes through infection and lysogenic conversion. mBio 2022; 13: e01851-22. 10.1128/mbio.01851-22 36154183 PMC9600943

[24] Partridge SR, Kwong SM, Firth N et al Mobile genetic elements associated with antimicrobial resistance. Clin Microbiol Rev 2018; 31: e00088-17. 10.1128/CMR.00088-17 30068738 PMC6148190

[25] Shang Y, Li D, Hao W et al A prophage and two ICESa2603-family integrative and conjugative elements (ICEs) carrying optrA in *Streptococcus suis*. J Antimicrob Chemother 2019; 74: 2876–9. 10.1093/jac/dkz309 31314095

[26] Koskella B, Meaden S. Understanding bacteriophage specificity in natural microbial communities. Viruses 2013; 5: 806–23. 10.3390/v5030806 PMC3705297 23478639

[27] Subramanian A . Emerging roles of bacteriophage-based therapeutics in combating antibiotic resistance. Front Microbiol 2024; 15: 1384164. 10.3389/fmicb.2024.1384164 39035437 PMC11257900

[28] Liu D, Van Belleghem JD, de Vries CR et al The safety and toxicity of phage therapy: a review of animal and clinical studies. Viruses 2021; 13: 1268. 10.3390/v13071268 PMC8310247 34209836

[29] Cohan FM, Zandi M, Turner PE. Broadscale phage therapy is unlikely to select for widespread evolution of bacterial resistance to virus infection. Virus Evol 2020; 6: veaa060. 10.1093/ve/veaa060 33365149 PMC7744382

[30] Upadhyay A, Jaiswal N, Kumar A. Biofilm battle: new transformative tactics to tackle the bacterial biofilm infections. Microb Pathog 2025; 199: 107277. 10.1016/j.micpath.2025.107277 39756524

[31] Liu S, Lu H, Zhang S et al Phages against pathogenic bacterial biofilms and biofilm-based infections: a review. Pharmaceutics 2022; 14: 427. 10.3390/pharmaceutics14020427 35214158 PMC8875263

[32] Abedon ST . Ecology and evolutionary biology of hindering phage therapy: the phage tolerance vs. phage resistance of bacterial biofilms. Antibiotics 2023; 12: 245. 10.3390/antibiotics12020245 PMC9952518 36830158

[33] Liu H, Wei X, Wang Z et al LysSYL: a broad-spectrum phage endolysin targeting *Staphylococcus* species and eradicating *S. aureus* biofilms. Microb Cell Fact 2024; 23: 89. 10.1186/s12934-024-02359-4 38528536 PMC10962180

[34] Sun X, Pu B, Qin J et al Effect of a depolymerase encoded by Phage168 on a carbapenem-resistant *Klebsiella pneumoniae* and its biofilm. Pathogens 2023; 12: 1396. 10.3390/pathogens12121396 38133282 PMC10745733

[35] Wang B, Du L, Dong B et al Current knowledge and perspectives of phage therapy for combating refractory wound infections. Int J Mol Sci 2024; 25: 5465. 10.3390/ijms25105465 PMC11122179 38791502

[36] Rahimzadeh Torabi L, Doudi M, Naghavi NS et al Isolation, characterization, and effectiveness of bacteriophage Pɸ-Bw-Ab against XDR *Acinetobacter baumannii* isolated from nosocomial burn wound infection. Iran J Basic Med Sci 2021; 24: 1254–63. 10.22038/ijbms.2021.57772.12850 PMC8751751 35083013

[37] Nadareishvili L, Hoyle N, Nakaidze N et al Bacteriophage therapy as a potential management option for surgical wound infections. PHAGE 2020; 1: 158–65. 10.1089/phage.2020.0010 36147826 PMC9041461

[38] Anastassopoulou C, Ferous S, Petsimeri A et al Phage-based therapy in combination with antibiotics: a promising alternative against multidrug-resistant Gram-negative pathogens. Pathogens 2024; 13: 896. 10.3390/pathogens13100896 39452768 PMC11510143

[39] Eskenazi A, Lood C, Wubbolts J et al Combination of pre-adapted bacteriophage therapy and antibiotics for treatment of fracture-related infection due to pandrug-resistant *Klebsiella pneumoniae*. Nat Commun 2022; 13: 302. 10.1038/s41467-021-27656-z 35042848 PMC8766457

[40] Loganathan A, Bozdogan B, Manohar P et al Phage-antibiotic combinations in various treatment modalities to manage MRSA infections. Front Pharmacol 2024; 15: 1356179. 10.3389/fphar.2024.1356179 PMC11041375 38659581

[41] Woolhouse M, Ward M, van Bunnik B et al Antimicrobial resistance in humans, livestock and the wider environment. Philos Trans R Soc Lond B Biol Sci 2015; 370: 20140083. 10.1098/rstb.2014.0083 25918441 PMC4424433

[42] Tang KL, Caffrey NP, Nóbrega DB et al Restricting the use of antibiotics in food-producing animals and its associations with antibiotic resistance in food-producing animals and human beings: a systematic review and meta-analysis. Lancet Planet Health 2017; 1: e316–27. 10.1016/S2542-5196(17)30141-9 PMC5785333 29387833

[43] Mulchandani R, Wang Y, Gilbert M et al Global trends in antimicrobial use in food-producing animals: 2020 to 2030. PLOS Glob Public Health 2023; 3: e0001305. 10.1371/journal.pgph.0001305 36963007 PMC10021213

[44] Johnson RP, Gyles CL, Huff WE et al Bacteriophages for prophylaxis and therapy in cattle, poultry and pigs. Anim Health Res Rev 2008; 9: 201–15. 10.1017/S1466252308001576 19102791

[45] Lang LH . FDA approves use of bacteriophages to be added to meat and poultry products. Gastroenterology 2006; 131: 1370. 10.1053/j.gastro.2006.10.012 17067600

[46] Colás-Medà P, Viñas I, Alegre I. Evaluation of commercial anti-listerial products for improvement of food safety in ready-to-eat meat and dairy products. Antibiotics 2023; 12: 414. 10.3390/antibiotics12020414 36830324 PMC9952070

[47] Imran A, Shehzadi U, Islam F et al Bacteriophages and food safety: an updated overview. Food Sci Nutr 2023; 11: 3621–30. 10.1002/fsn3.3360 37457180 PMC10345663

[48] Ranveer SA, Dasriya V, Ahmad MF et al Positive and negative aspects of bacteriophages and their immense role in the food chain. NPJ Sci Food 2024; 8: 1. 10.1038/s41538-023-00245-8 38172179 PMC10764738

[49] EFSA Panel on Biological Hazards (BIOHAZ) . Evaluation of the safety and efficacy of Listex^TM^ P100 for reduction of pathogens on different ready-to-eat (RTE) food products. EFSA J 2016; 14: e04565. 10.2903/j.efsa.2016.4565

[50] Everhart E, Worth A, D’Amico DJ. Control of *Salmonella enterica* spp. enterica in milk and raw milk cheese using commercial bacteriophage preparations. Food Microbiol 2025; 128: 104725. 10.1016/j.fm.2025.104725 39952766

[51] Pires DP, Costa AR, Pinto G et al Current challenges and future opportunities of phage therapy. FEMS Microbiol Rev 2020; 44: 684–700. 10.1093/femsre/fuaa017 32472938

[52] Zalewska-Piątek B . Phage therapy—challenges, opportunities and future prospects. Pharmaceuticals (Basel) 2023; 16: 1638. 10.3390/ph16121638 PMC10747886 38139765

[53] Voelker R . FDA approves bacteriophage trial. JAMA 2019; 321: 638. 10.1001/jama.2019.0510 30778586

[54] Karn SL, Gangwar M, Kumar R et al Phage therapy: a revolutionary shift in the management of bacterial infections, pioneering new horizons in clinical practice, and reimagining the arsenal against microbial pathogens. Front Med 2023; 10: 1209782. 10.3389/fmed.2023.1209782 PMC10620811 37928478

[55] Pelfrene E, Willebrand E, Cavaleiro Sanches A et al Bacteriophage therapy: a regulatory perspective. J Antimicrob Chemother 2016; 71: 2071–4. 10.1093/jac/dkw083 27068400

[56] Yang Q, Le S, Zhu T et al Regulations of phage therapy across the world. Front Microbiol 2023; 14: 1250848. 10.3389/fmicb.2023.1250848 37869667 PMC10588630

[57] Products | R&D and production. MB Pharma n.d. https://www.mbph.cz/produkty?lang=en.

[58] Phage products—BioChimPharm. https://biochimpharm.ge/en/products/.

[59] Phago-FERSISI bacteriophage. Phage n.d. https://phage.ge/en/products/phago-fersisi.

[60] Phago-Staph bacteriophage. Phage n.d. https://phage.ge/en/products/phago-staph.

[61] Phago-Pyo. Phage n.d. https://phage.ge/en/products/phago-pyo.

[62] Phago-Intesti bacteriophage. Phage n.d. https://phage.ge/en/products/phago-intesti.

[63] Phago-SES bacteriophage. Phage n.d. https://phage.ge/en/products/phago-ses.

[64] Phago-ENKO bacteriophage. Phage n.d. https://phage.ge/en/products/phago-enko.

[65] Bacteriophages n.d. https://www.microgen.ru/en/products/bakteriofagi/.

[66] Phagogyn—RPC «Micromir» n.d. https://micromir.bio/products/fagogin.

[67] Phagodent—RPC «Micromir» n.d. https://micromir.bio/products/fagodent.

[68] Phagoderm—RPC «Micromir» n.d. https://micromir.bio/products/fagoderm.

[69] Otophage—RPC «Micromir» n.d. https://micromir.bio/products/otofag.

[70] Iskraphage—RPC «Micromir» n.d. https://micromir.bio.

[71] Phagex n.d. https://bacteriophages.info/en/bacteriophage/.

[72] Fauconnier A . Phage therapy regulation: from night to dawn. Viruses 2019; 11: 352. 10.3390/v11040352 30999559 PMC6521264

[73] Kaistha SD, Devi P, Sharma N et al Navigating regulatory frameworks and compliances for bacteriophagesas therapeutic agents. Curr Pharm Biotechnol 2025; 26: 1–12. 10.2174/0113892010347488250113171505 39844559

[74] Kushwaha SO, Sahu SK, Yadav VK et al Bacteriophages as a potential substitute for antibiotics: a comprehensive review. Cell Biochem Funct 2024; 42: e4022. 10.1002/cbf.4022 38655589

[75] Fischetti VA . Development of phage lysins as novel therapeutics: a historical perspective. Viruses 2018; 10: 310. 10.3390/v10060310 PMC6024357 29875339

[76] Fischetti VA . Bacteriophage endolysins: a novel anti-infective to control gram-positive pathogens. Int J Med Microbiol 2010; 300: 357–62. 10.1016/j.ijmm.2010.04.002 20452280 PMC3666336

[77] Fowler VG, Das AF, Lipka-Diamond J et al Exebacase for patients with *Staphylococcus aureus* bloodstream infection and endocarditis. J Clin Invest 2020; 130: 3750–60. 10.1172/JCI136577 32271718 PMC7324170

[78] Zhang H, Zhang X, Liang S et al Bactericidal synergism between phage endolysin Ply2660 and cathelicidin LL-37 against vancomycin-resistant *Enterococcus faecalis* biofilms. NPJ Biofilms Microbiomes 2023; 9: 16. 10.1038/s41522-023-00385-5 PMC10078070 37024490

[79] Dicks LMT, Vermeulen W. Bacteriophage–host interactions and the therapeutic potential of bacteriophages. Viruses 2024; 16: 478. 10.3390/v16030478 38543843 PMC10975011

[80] Khosravi A, Chen Q, Echterhof A et al Phage therapy for respiratory infections: opportunities and challenges. Lung 2024; 202: 223–32. 10.1007/s00408-024-00700-7 38772946 PMC11570333

[81] Viertel TM, Ritter K, Horz H-P. Viruses versus bacteria-novel approaches to phage therapy as a tool against multidrug-resistant pathogens. J Antimicrob Chemother 2014; 69: 2326–36. 10.1093/jac/dku173 24872344

[82] Panagiotopoulos A-P, Sagona AP, Tsakri D et al Virological and pharmaceutical properties of clinically relevant phages. Antibiotics 2025; 14: 487. 10.3390/antibiotics14050487 40426553 PMC12108485

[83] Gan T, Wang D. Picobirnaviruses encode proteins that are functional bacterial lysins. Proc Natl Acad Sci U S A 2023; 120: e2309647120. 10.1073/pnas.2309647120 37669381 PMC10500164

[84] Krishnamurthy SR, Wang D. Extensive conservation of prokaryotic ribosomal binding sites in known and novel picobirnaviruses. Virology 2018; 516: 108–14. 10.1016/j.virol.2018.01.006 29346073

[85] Hietala V, Horsma-Heikkinen J, Carron A et al The removal of endo- and enterotoxins from bacteriophage preparations. Front Microbiol 2019; 10: 1674. 10.3389/fmicb.2019.01674 31396188 PMC6664067

